# Improved Quantification of ^18^F-FDG PET during ^131^I-Rituximab Therapy on Mouse Lymphoma Models after ^131^I Prompt Emission Correction

**DOI:** 10.3390/diagnostics9040144

**Published:** 2019-10-08

**Authors:** Young Sub Lee, Hee-Joung Kim, Jin Su Kim

**Affiliations:** 1 Division of RI Application, Korea Institute Radiological and Medical Sciences, Seoul 01812, Korea; yslee@kins.re.kr; 2 Division of Radiation Regulation, Department of Medical Radiation Safety, Korea Institute of Nuclear Safety, Daejeon 34142, Korea; 3 Department of Radiation Convergence Engineering and Research Institute of Health Science, Yonsei University, Wonju 26493, Korea; hjk1@yonsei.ac.kr; 4 Radiological and Medico-Oncological Sciences, University of Science and Technology, Seoul 01812, Korea

**Keywords:** ^18^F-FDG PET, personalized medicine, ^131^I prompt emission-correction, radioimmunotherapy, rituximab, rituxan

## Abstract

^18^F-FDG Positron Emission Tomography (PET) is used to monitor tumor response to ^131^I-therapy, but is confounded by prompt emissions (284, 364, 637, and 723 keV) from ^131^I, particularly in animal PET imaging. We propose a method for correcting this emission in ^18^F-FDG PET. The ^131^I prompt emission effect was assessed within various energy windows and various activities. We applied a single gamma correction method to a phantom and in vivo mouse model. The ^131^I prompt emission fraction was 12% when 300 µCi of ^131^I and 100 µCi of FDG were administered, and increased exponentially with escalating ^131^I activity for all energy windows. The difference in spill-over ratio was reduced to <5% after ^131^I prompt emission correction. In the mouse model, the standard uptake value (SUV) did not differ significantly between FDG PET only (gold standard) and FDG PET after ^131^I prompt emission-correction, whereas it was overestimated by 38% before correction. Contrast was improved by 18% after ^131^I prompt emission correction. We first found that count contamination on ^18^F-FDG follow-up scans due to ^131^I spilled-over count after ^131^I rituximab tumor targeted therapy. Our developed ^131^I prompt emission-correction method increased accuracy during measurement of standard uptake values on ^18^F-FDG PET.

## 1. Introduction

Radioimmunotherapy (RIT), such as ^131^I labelled tositumomab (Bexxar) and ^131^I labelled rituximab [1,2], is used for targeted treatment of cancer and involves selective delivery of radionuclide-labelled monoclonal antibodies (mAbs) [1,3]. Imaging mAb using Positron Emission Tomography (PET) or Single-photon emission computed tomography (SPECT) was applied to quantitatively estimate expression of accessible antigens in the target tissue [4,5,6]. A gamma camera or SPECT imaging was used for ^131^I imaging [7]. A conventional gamma camera is limited in terms of detection of differentiated thyroid carcinoma, owing to the biokinetics in the lesion and background in individual patients [8]. ^124^I PET has been used to image the residual thyroid lesion after ^131^I thyroid therapy [9] and can provide the dosimetry data for the activity of ^131^I administered for therapy of differentiated thyroid cancer [10]. The ^124^I PET-based response rates of small lymph node metastases and thyroid remnants in a minimum high-absorbed radiation dose group matched the histological data after administration of therapeutic ^131^I [11]. ^124^I PET imaging for the assessment of ^131^I therapy was superior to the use of ^18^F-FDG PET because ^124^I and ^131^I had the same chemical properties due to their isotope relationship. However, conventional ^18^F-FDG PET was still used for monitoring of the therapeutic effect of ^131^I [12], this was due to the easy accessibility of ^18^F-FDG PET in clinics. For example, the use of ^18^F-FDG PET was reported for the short temporal response of Hodgkin’s disease to RIT [13]. The clinical significance of ^18^F-FDG uptake by primary sites in patients with diffuse large B cell lymphoma in the head and neck, or in cervical lymph nodes, was reported [14,15]. The use of ^18^F-FDG PET imaging of early response to predict prognosis in the first-line management of follicular non-Hodgkin lymphoma with ^131^I Rituximab RIT was reported [16]. The value of ^18^F-FDG PET/CT for the staging of primary extranodal head and neck lymphomas was also reported [17]. In clinics, the early response to a therapy was performed about 12 weeks after administration of ^131^I rituximab. However, in a preclinical mouse model, tumor size increases faster than in humans, while their maximum life span is much shorter than humans. The tumor in lymphoma mouse model grew from 200 mm^3^ to 100 mm^3^ within 10 days. In preclinical study, therefore, there was a necessity of a ^18^F-FDG PET follow-up scan immediately after ^131^I therapy.

^131^I emits gamma rays (284 keV (6%), 364 keV (82%), 637 keV (7%), and 723 keV (2%)) and beta rays (334 keV (7%), and 606 keV (90%)) for therapy. When ^131^I rituximab therapy is monitored using ^18^F-FDG PET, findings may be contaminated by the inclusion of prompt emissions (364 keV (82%) and 637 keV (7.16%)) from ^131^I. This phenomenon is particularly prominent during animal PET, because wider energy windows, such as 250–750 keV or 350–750 keV, are used to increase the sensitivity of animal PET imaging. The high energy of the most abundant gammas can cause down scatter issues within the energy window of the 511 keV annihilation photons of PET imaging. Whether the low energy due to ^131^I could also contaminate ^18^F-FDG PET scans is unknown. The effect of ^131^I prompt emission required assessment, because 364 keV and 637 keV emissions from gamma photons are within the conventional PET acquisition energy windows. During a ^18^F-FDG follow-up study, we should check the possibility of inclusion of ^131^I within PET acquisition.

The motivation of our study was the necessity of a quantification method of ^18^F-FDG PET during ^131^I therapy. To the best of our knowledge, there has been no report on the assessment of ^131^I prompt emission or the development of ^131^I prompt emission-correction for ^18^F-FDG PET imaging. Our data showed that our ^131^I prompt emission-correction method was feasible for use in ^18^F-FDG follow-up after ^131^I therapy in a preclinical study.

This study aimed to assess the count contamination on ^18^F-FDG follow-up scans due to ^131^I spill-over count after ^131^I rituximab tumor targeted therapy. We measured the effect of ^131^I prompt emission with various activity levels and energy windows using a phantom and animal model. We found count contamination on ^18^F-FDG follow-up scans due to ^131^I spill-over count after ^131^I rituximab tumor targeted therapy. To limit this contamination, we developed the ^131^I prompt emission correction method during ^18^F-FDG PET.

## 2. Materials and Methods

In this study, we identified the effect of ^131^I therapy during FDG PET scanning. For this purpose, we performed GATE Monte Carlo simulation, phantom study, and actual in vivo study using lymphoma mouse model. All mice-related experiments were performed under a protocol approved by IACUC (number KIRAMS 2013-104, date of approval: 6 January 2014) of the Korea Institute of Radiological and Medical Sciences (KIRAMS).

### 2.1. ^131^I Prompt Emissions during PET

**Figure 1:**
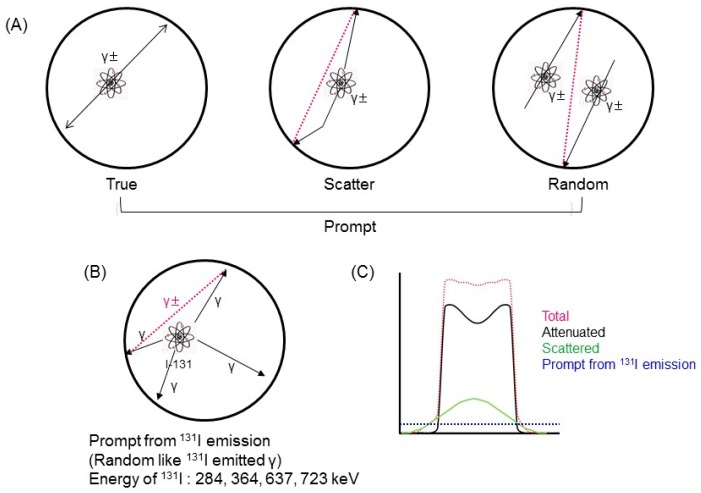
Schematic illustration of emissions. (**A**) Schematic of Positron Emission Tomography (PET) emissions, such as true, scatter, and random emissions during annihilation. (**B**) ^131^I prompt emission. ^131^I emitted 284, 364, 637, and 723 keV gamma rays. (**C**) Schematic of ^131^I prompt emission profile.

Figure 1A shows a schematic illustration of PET emissions, such as true, scatter, and random emissions during annihilation. Figure 1B shows the prompt emissions due to ^131^I. ^131^I emitted 284, 364, 637, and 723 keV gamma rays. We previously developed a method for correction of ^124^I single gamma ray emissions, such as those at 622 and 723 keV [18,19]. ^124^I prompt emissions create background noise [20] (shown in Figure 2 in [20]). The characteristics of ^131^I prompt emissions were similar to those of ^124^I. Therefore, ^131^I prompt emissions were also distributed as background noise (Figure 1C). A Siemens Inveon PET scanner (Siemens, Erlangen, Germany) was used for monitoring.

### 2.2. GATE Simulation

We calculated the fraction of ^131^I prompt emissions due to ^131^I by GATE simulation, with various energy windows and various activity levels. We applied our ^131^I prompt emission-correction method to ^18^F-FDG PET in both a phantom and animal study.

### 2.3. Estimation of Prompt Emission Counting Rate due to A Single Gamma Photon from ^131^I

The prompt emission counting rate was estimated using GATE simulation. The prompt emission due to ^131^I would increase with increasing activities of ^131^I. Therefore, activities were set from 100 µCi to 1000 µCi in steps of 100 µCi. The energy windows were 250–650 keV, 250–750 keV, 350–650 keV, 350–750 keV, 450–650 keV, and 450–750 keV, respectively. A lower energy level discriminator setting of 350 keV and 450 keV was used to assess the effect of 364 keV ^131^I prompt emissions. An upper energy level discriminator setting of 750 keV was used to assess the effect of 637 keV ^131^I prompt emissions. To estimate the prompt emission counting rate due to ^131^I prompt emissions in GATE simulation, the output file was saved in ASCII format. The ASCII output file was an interfile with coded integers in 4 bytes without a header. The prompt emission counting rate due to ^131^I prompt emissions was extracted from the ASCII file.

### 2.4. ^131^ I Prompt Emission Fraction

When ^18^F-FDG and ^131^I were imaged simultaneously, ^131^I prompt emission would contaminate ^18^F-FDG PET images. The effect of count contamination due to ^131^I prompt emission would escalate as ^131^I activity increases. In this study, the ^131^I prompt emission fraction was defined as “^131^I prompt emission / (^18^F prompt + ^131^I prompt emission)”. The activity of ^18^F-FDG was set to 100 µCi and the ^131^I activities of 1 µCi, 10 µCi, 100 µCi to 1000 µCi (in steps of 100 µCi), and 10 mCi, were assessed.

### 2.5. Correction of ^131^I Prompt Emission on ^18^F-FDG PET Imaging

The distribution of ^131^I prompt gamma was nearly uniform because the angle of emission of the prompt gamma was uncorrelated with the angle of the annihilation photons [21,22]. For the correction of ^131^I prompt gamma, first, the scatter sinogram was generated using a single scatter simulation scatter correction algorithm [23]. The edge of the scatter sinogram was identified by thresholding. The data outside the body was then calculated. ^131^I prompt gamma was calculated using following equation.

^131^I prompt gamma signogram = scatter sinogram × scale factor (1)

where scale factor = ^131^I prompt emission fraction.

The prompt gamma and scatter distributions were subtracted from the uncorrected sinogram. The ^131^I prompt emission-corrected sinogram was reconstructed with correction processes, such as attenuation, scatter, deadtime correction, and normalization. The rebinning and reconstruction were performed using the IAW program (ver. 1.4.3.6) provided by Siemens (Erlangen, Bavaria, Germany).

### 2.6. Phantom Study (Spill-Over Ratio)

^131^I prompt emission-correction was applied to a phantom image. A NEMA NU4-2008 image-quality phantom was used to validate the prompt emission-photon correction. The activities were 1 mCi for ^131^I and 100 μCi for ^18^F-FDG. PET data were acquired for 20 min of emission and 15 min of transmission using an INVEON PET scanner. Transmission data were acquired using a ^57^Co source within an energy window of 120–125 keV. The energy window of the emission scan was 350–650 keV. PET data were reconstructed with filtered back-projection (FBP) algorithms. To assess the effect of ^131^I prompt emission-correction, image quality was assessed in terms of the spill-over ratio (SOR) according to the NEMA NU4-2008 guidelines. The SOR was defined as the ratio between the mean value of a cold cylinder and the mean value of the uniform area. The upper part of the uniform region was a cold region consisting of two empty cylinders (length: 15 mm, inner diameter: 8 mm, outer diameter: 10 mm). One space was filled with air and the other space with nonradioactive water. To calculate the SOR, 2 cylindrical VOIs (length: 7.5 mm, diameter: 4 mm) were drawn in the air- and water-filled compartments. We compared the SOR before and after prompt emission-photon correction. For the phantom study, we compared the results of pre- and post-^131^I prompt emission fraction correction for a NEMA NU4-2008 image quality phantom [24]. To calculate the SOR, the volumes of interest (VOIs) were drawn on the nonradioactive region using a diameter 75% of that of the nonradioactive region. PET data were reconstructed using FBP. Normalization, dead-time, attenuation and scatter corrections have been applied to all PET raw data. However, partial volume correction was not applied during measurement of SOR because diameter of water filled- and air filled cylinder was relatively large (length: 15 mm, inner diameter: 8 mm, outer diameter: 10 mm) to avoid the partial volume effect. 

### 2.7. In Vivo Study

#### 2.7.1. Lymphoma Animal Model

We applied our developed prompt emission-photon-correction method to a mouse lymphoma model. To generate the mouse lymphoma model, human Burkitt CD20+ (Raji) cells were obtained from the American Type Culture Collection (Manassas, VA, USA), and maintained in roswell park memorial istitute (RPMI) medium containing 10% fetal bovine serum and antibiotics (Sigma, St Louis MO, USA). Cells were kept at 37 °C in a humidified 5% CO_2_ incubator. Raji cells (1 × 10^7^) were subcutaneously injected into female NOD/SCID mice (*n* = 10) (Animal Resource Centre, Murdoch, WA, Australia). The tumor volume was calculated using the formula (width^2^ × length × 0.4).

#### 2.7.2. Preparation of ^131^I Rituximab

Precoated iodination tubes (Thermo Scientific, Waltham, MA, USA) were used for preparing ^131^I rituximab for treating the lymphoma model mice. For radiolabeling, the Pierce precoated iodination tube was wet with 1 mL Tris iodination buffer, which was then again decanted, after which 60 µL (1.0 mCi) of ^131^I was added to the iodination tube. Iodide was activated for 6 min at room temperature, then, 200 μg of rituximab was added and reacted with the iodide for 6–9 min at room temperature. Instant thin-layer chromatography (solvent: 100% acetone, C_3_H_6_O) showed that ^131^I rituximab had a radiochemical purity >95%.

#### 2.7.3. PET Imaging of Lymphoma Mouse Model

After confirmation of the ^131^I rituximab uptake to tumor region, we made additional lymphoma tumor model (*n* = 5). When the tumor size reached 300 mm^3^, an ^18^F-FDG PET scan of the lymphoma model was acquired as a gold standard to assess the standard uptake value (SUV) before administration of ^131^I rituximab. After 10 half-lives of ^18^F-FDG, ^131^I rituximab was administered intravenously. After 48 h, ^18^F-FDG PET was acquired. The activity of ^131^I rituximab was 300 µCi/75 mg. The activity of ^18^F-FDG was 100 µCi. PET data was reconstructed using FBP. The ^131^I prompt emission-correction was applied to the data. All corrections, such as attenuation correction, scatter correction, dead-time correction, and normalization was performed. To assess the effect of ^131^I prompt emission, an ROI (20–30 mm^2^) was drawn on the tumor region in reconstruction trans-axial PET images both before and after ^131^I prompt emission-correction. Contrast was also assessed within the tumor region. An ROI was drawn on the necrotic area in a cold region and on the tumor area in a hot region. Contrast was defined as the SUV of the hot region/the SUV of the cold region. The maximal value of SUV was measured for minimizing partial volume effect.

## 3. Results

The GATE code for the Inveon PET scanner simulation was modelled in our previous study [25]. The photoelectric effect, as well as Compton and Rayleigh interactions, were modelled. Energy cuts were performed on all simulated models. Energy blurring was set to 11% resolution. All physical processes of emission and interaction for ^131^I and ^18^F point sources were simulated.

**Figure 2:**
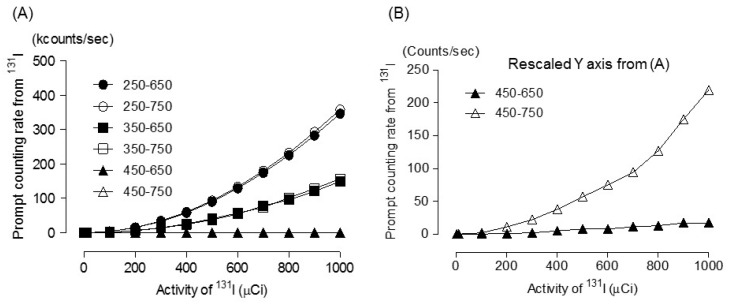
(**A**) Prompt emission counting rate due to ^131^I within various energy windows. (**B**) Rescaled Y axis from Figure 2A. The value between “450 and 650 keV” and “450 and 750 keV” was not discernible in the scale of Kcounts/sec in Figure 2A. Therefore, we rescaled the Y-axis for the visibility of “450–650 keV” and “450–750 keV” in Figure 2B.

Figure 2 shows the Monte Carlo simulation result of the prompt emission counting rate for ^131^I during PET scans using various PET energy windows. Although the prompt emission counting rate at 300 µCi ^131^I was <15 kcps, the prompt emission counting rate reached 149 kcps and 10,117 kcps (not shown in graph) when the activity was 1000 µCi and 10,000 mCi ^131^I, respectively, within a window of 350–650 keV. However, the prompt emission counting rate, even at 1000 µCi ^131^I was nearly 0 (2 cps) within a window of 450–650 keV. This result demonstrated that 364 keV and 637 keV of a prompt emission-photon from ^131^I was not influential within the 450–650 keV windows.

**Figure 3:**
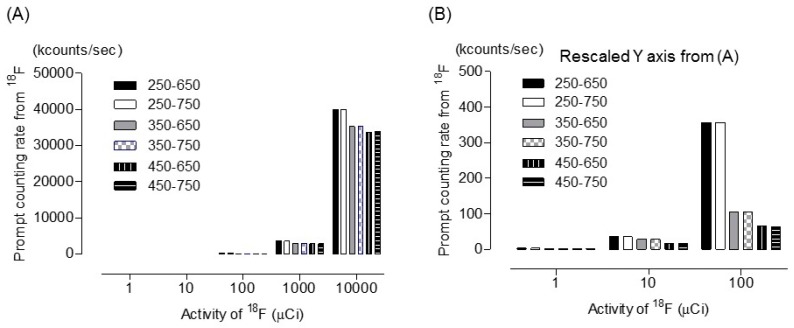
(**A**) Prompt emission counting rate due to ^18^F within various energy windows. (**B**) Rescaled Y axis from Figure 3A. Activities were set to 1, 10, 100, 100, 10,000 µCi.

**Figure 4:**
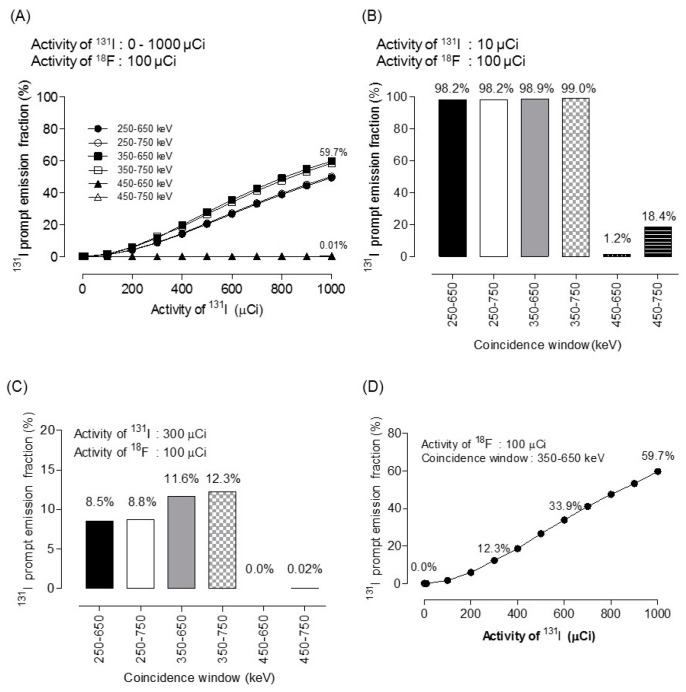
^131^I prompt emission fraction within various energy windows. (**A**) Activity of ^131^I was set to 0 to 1000 µCi in steps of 100 µCi with 100 µCi ^18^F activity. (**B**) 10 µCi of ^131^I with 100 µCi of ^18^F. (**C**) 300 µCi of ^131^I with 100 µCi of ^18^F (**D**) ^131^I prompt emission fraction within 350–650 keV at the level of 100 µCi of ^18^F.

Figure 3 shows the prompt emission counting rate for ^18^F. Activities were set to 1, 10, 100, 100, and 10,000 µCi. A lower energy level discriminator setting of 350 keV and 450 keV was used to assess the effect of 364 keV of ^131^I prompt emission. An upper energy level discriminator setting of 650 keV and 750 keV was used to assess the effect of 637 keV and 723 keV of ^131^I prompt emission. Although the prompt emission counting rate at 300 µCi of ^131^I was <15 kcps, the coincidence reached 157 kcps and 10 Mcps when the activity was 1 mCi and 10 mCi of ^131^I within 350–650 keV, respectively. However, the coincidence rate with 300 µCi ^131^I was nearly 0 (2 cps) within the 450–650 keV window. This demonstrated that the ^131^I prompt emission at 364 keV and 637 keV had been discarded within the 450–650 keV window. Table 1 and Figure 4 shows the ^131^I prompt emission fraction within various energy windows. Figure 4D shows that, within a 350–650 keV window, the ^131^I prompt emission fraction was 12% when 300 µCi ^131^I and 100 µCi ^18^F-FDG were administered. The ^131^I prompt emission fraction reached 59.7% and 99% when 1 mCi 131 I and 10 mCi ^131^I were administered, respectively. The ^131^I prompt emission fraction increased with increasing ^131^I activity for all energy windows. The relationship between the ^131^I prompt emission fraction and ^131^I activity within the 350–650 keV window was as follows:

^131^I prompt emission fraction = 0.06 × (activity of ^131^I [µCi]) − 6.72 (*R*^2^ = 0.99) (2)

The ^131^I prompt emission fraction was defined as the “coincidence from ^131^I/(coincidence from ^18^F-FDG + coincidence from ^131^I)”. The ^131^I prompt emission fraction was 12% when 300 µCi of ^131^I and 100 µCi of ^18^F-FDG were co-administered. ^131^I prompt emission fraction reached 59.7% when 1 mCi of ^131^I was administered.

To correct ^131^I prompt emission, we applied our developed ^131^I prompt emission-correction method to both a phantom and an in vivo mouse lymphoma model.

**Figure 5:**
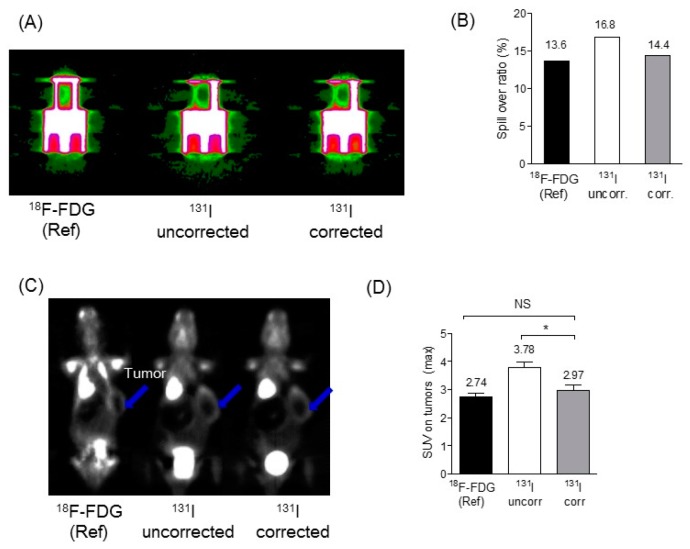
(**A**) Positron Emission Tomography (PET) image of NEMA image quality phantom, (**B**) spill-over ration of NEMA image quality phantom, (**C**) Representative PET data of mouse image, arrow sign indicates tumor region, (**D**) SUV in tumors, * *p* < 0.05.

Figure 5A shows the representative PET image of NEMA NU4-2008 image quality phantom and Figure 5B shows the SOR results compared with the gold standard (^18^F-FDG), before ^131^I prompt emission and after ^131^I prompt emission. SOR was 13.7% for ^18^F-FDG PET. After administration of ^131^I, SOR was 16.9% and 14.4% before and after ^131^I prompt emission correction, respectively. The percentage difference was <5% between ^18^F-FDG only and after ^131^I prompt emission-correction.

When our ^131^I prompt emission-correction method was applied to the in vivo mouse model (shown in Figure 5C), the SUV of the tumor region was 2.74 ± 0.13 for ^18^F-FDG PET only, 2.97 ± 0.18 after ^131^I prompt emission-correction, and 3.78 ± 0.2 before ^131^I prompt emission-correction (shown in Figure 5D. Contrast was improved by 18% after ^131^I prompt emission-correction. Before ^131^I prompt emission-correction, SUV was overestimated by 38% (* *p* < 0.05). However, there was no statistically significant difference between SUV for ^18^F-FDG and after correction of ^131^I prompt emission.

## 4. Discussion

Prompt correction method of nonpure positron emitters including ^82^Rb [21,22], ^124^I [18,26,27], ^76^Br [28,29], and ^86^Y [29] were introduced. This prompt gamma can directly contribute or indirectly scatter down into the primary energy window [26]. The high energy of gammas can cause down scatter issues within the energy window of the 511 keV annihilation photons of PET imaging. We investigated whether low energy due to ^131^I could also contaminate ^18^F-FDG PET scans. We found count contamination on ^18^F-FDG follow-up scans due to ^131^I spill-over count after ^131^I rituximab tumor targeted therapy. For this reason, we developed a PET image correction method to address the inclusion of ^131^I during ^18^F-FDG PET scans. When our developed method was applied to the measurement of SOR using the NEMA NU4 image quality phantom, ^131^I prompt correction provided the similar level of SOR (the percentage difference was <5% between ^18^F-FDG only and after ^131^I prompt emission-correction). When our ^131^I prompt emission-correction method was applied to the in vivo mouse model, the SUV of the tumor region was 2.74 ± 0.13 for ^18^F-FDG PET only, 2.97 ± 0.18 after ^131^I prompt emission-correction, and 3.78 ± 0.2 before ^131^I prompt emission-correction. We found that our developed ^131^I prompt correction method was applicable for both phantom and actual mouse study. Our ^131^I prompt emission-correction method increased accuracy during measurement of standard uptake value on ^18^F-FDG PET as well as spill-over ratio in phantom study.

We assessed the effect of ^131^I in terms of SOR in a phantom and also applied it to a mouse model that had received ^131^I rituximab. Our data showed that count contamination due to ^131^I prompt emission was prominent when ^131^I at higher activities was administered (at 10 mCi ^131^I, the ^131^I prompt emission fraction reached 99%). In addition, we found that there was a negligible effect of ^131^I prompt emission within an energy window of 450–650 keV. Therefore, this energy window would be useful for ^18^F-FDG follow-up scans. However, in a narrow energy window, such as 450–650 keV, the sensitivity would be significantly decreased when the conventional 350–650 keV energy window is used in a preclinical study. According to our Monte Carlo simulation study for the Inveon PET scanner, the sensitivities were 7.0% for 350–650 keV, 7.3% for 350–750 keV, 5.8% for 450–650 keV, and 6.0% for 450–750 keV windows. The sensitivity within the 450–650 keV window was degraded by 26% as compared to that of the 350–650 keV window. Decreased sensitivity in a PET scan indicates a decreased signal-to-noise ratio in PET. Therefore, there was a trade-off between count contamination due to ^131^I prompt emissions and the signal-to-noise ratio between a narrow energy window (450–650 keV) and the conventional energy window (350–650 keV) in ^18^F-FDG PET after ^131^I administration. With application of our ^131^I prompt correction method, a wider energy window, such as 350–650 keV would be advantageous over a 450–650 keV energy window.

The main limitation of this study was, given that our developed method was applicable to animal study, we used a wide energy window during PET scanning to improve sensitivity. However, narrower energy windows are typically used in a clinical setting and a low energy of ^131^I would be negligible during ^18^F-FDG scanning. In summary, although these findings have a limited application for clinical use, our developed method could be feasible in animal studies, especially in ^18^F-FDG PET assessment of the therapeutic efficacy of RIT, such as ^131^I rituximab.

## 5. Conclusions

In conclusion, we developed an ^131^I prompt emission-correction method for ^18^F-FDG PET imaging after ^131^I rituximab therapy and applied it to an in vivo mouse model. Our method will facilitate monitoring of the therapeutic efficacy of newly developed drugs in ^18^F-FDG PET follow-up after ^131^I-rituximab therapy.

## Figures and Tables

**Table 1:** ^131^I prompt emission fraction with various energy windows and activities.

Activity of ^131^I * (µCi)	Energy Window (keV)			
	350–650	350–750	450–650	450–750
1	0.00	0.00	0.00	0.00
10	0.01	0.01	0.00	0.00
100	1.45	1.60	0.00	0.00
200	5.59	5.88	0.00	0.01
300	11.64	12.25	0.00	0.02
400	19.70	18.65	0.00	0.04
500	27.85	26.53	0.01	0.05
600	35.31	33.92	0.01	0.07
700	42.53	41.12	0.01	0.09
800	49.08	47.54	0.01	0.12
900	54.81	53.30	0.02	0.17
1000	58.50	59.74	0.02	0.21
10,000	98.96	99.01	1.28	18.48

* In this GATE simulation for the calculation of the ^131^I prompt emission fraction, the activity of ^18^F was set to 100 µCi.
